# Unilateral Compound Training Reduces Lower-Limb Strength Asymmetry and Enhances Athletic Performance in Female Handball Players: A Randomized Controlled Trial

**DOI:** 10.3390/life16071169

**Published:** 2026-07-15

**Authors:** Erkan Güven, Gizem Akarsu Taşman, Nasuh Evrim Acar, Bilal Gök, Zarife Pancar

**Affiliations:** 1 Faculty of Sports Sciences, Mersin University, Mersin 33110, Türkiye; 2 Exercise and Sports Sciences Application and Research Center, Mersin University, Mersin 33110, Türkiye; 3 Faculty of Sports Sciences, Istanbul Gelişim University, Istanbul 34310, Türkiye; 4 Department of Physical Education and Sports, Faculty of Sports Science, Gaziantep University, Gaziantep 27350, Türkiye

**Keywords:** handball, unilateral training, strength asymmetry, isokinetic strength, plyometric training, athletic performance

## Abstract

Lower-limb strength asymmetries are common in handball players and may negatively influence athletic performance while increasing injury risk. This study examined the effects of a six-week unilateral compound training program on lower-limb strength asymmetry, isokinetic strength, the hamstring-to-quadriceps (H/Q) ratio, body composition, and physical performance in competitive female handball players. Thirty highly trained female handball athletes (age: 18.75 ± 1.91 years) were randomly assigned to an experimental group (*n* = 14) or a control group (*n* = 16). The experimental group completed a unilateral compound training program consisting of unilateral resistance and plyometric exercises three times per week for six weeks, in addition to regular handball training, whereas the control group continued regular training only. Before and after the intervention, participants underwent assessments of body composition, isokinetic knee strength, bilateral strength asymmetry, H/Q ratios, 20 m sprint performance, change-of-direction (COD) ability, countermovement jump (CMJ), and single-leg CMJ performance. Significant group × time interaction effects were observed for body fat percentage, muscle percentage, muscle mass, knee flexor strength (both limbs), left knee extensor strength, bilateral quadriceps and hamstring asymmetries, H/Q ratios, sprint performance, COD performance, CMJ height, and single-leg jump performance (*p* < 0.05); the interaction for right knee extensor strength did not reach statistical significance (*p* = 0.072). The experimental group’s improvements coincided with substantial reductions in quadriceps (13.19% to 7.60%; −42.38%) and hamstring (16.65% to 7.83%; −52.97%) asymmetries, alongside changes in H/Q ratios, isokinetic strength, sprint performance (−4.68%), COD performance (−5.91%), CMJ height (+12.56%), and single-leg jump performance (+11.42–18.36%; partial η^2^ = 0.19–0.46 across these primary outcomes). Given the relatively modest sample size, these percentage reductions should be interpreted with appropriate caution, as they may be subject to overestimation. Because the experimental group received three additional weekly training sessions relative to the control group, the observed improvements cannot be unambiguously attributed to the unilateral training modality itself, as opposed to the additional training volume. These findings suggest that adding a supplemental unilateral compound training program to regular handball training may improve lower-limb strength asymmetry and neuromuscular performance. However, because the intervention also increased total training volume, these effects cannot be attributed exclusively to the unilateral training modality itself.

## 1. Introduction

Handball is a high-intensity intermittent team sport characterized by repeated bouts of sprinting, jumping, rapid changes in direction, physical contact, and explosive throwing actions performed under conditions of considerable physiological and neuromuscular stress [1,2]. During competition, players are required to repeatedly execute high-intensity movements such as accelerations, decelerations, jumps, landings, and multidirectional actions while maintaining technical and tactical efficiency throughout the match [3,4]. Notably, many of these sport-specific actions are performed unilaterally, placing substantial mechanical demands on the lower extremities and increasing the likelihood of asymmetrical loading patterns between limbs [5,6]. Given that these unilateral actions are central to handball performance, training interventions that similarly emphasize unilateral loading patterns may be particularly well suited to address the specific neuromuscular demands and asymmetries inherent to the sport, in contrast to traditional bilateral training approaches, which may not adequately replicate these sport-specific movement patterns or their associated inter-limb loading demands. Consequently, optimizing lower-limb neuromuscular function represents a critical objective for coaches and practitioners aiming to enhance performance while minimizing injury risk in handball athletes [1,2].

Given the high neuromuscular demands of handball, the development of lower-limb strength and power is considered a fundamental component of athletic preparation. To enhance these physical qualities, coaches frequently incorporate resistance and plyometric training into their conditioning programs [7]. While both training modalities have independently demonstrated positive effects on muscular strength, power output, sprinting ability, and jumping performance, accumulating evidence suggests that their combined application may induce superior adaptations compared with either method performed in isolation [8]. This training approach, commonly referred to as compound training, integrates a full block of resistance exercises with a full block of plyometric exercises within the same session, separated by a recovery period, and aims to maximize neuromuscular adaptations through the interaction of force- and velocity-oriented stimuli [9]. This differs from complex training, in which a heavy resistance exercise is paired immediately with a biomechanically similar plyometric exercise to exploit post-activation potentiation [9]. Compound training was selected for the present population because it allows greater total training volume within a session, is more feasible in team-sport settings with limited rest periods, and does not require the precise exercise pairing and timing that complex training demands. Such adaptations are believed to enhance the efficiency of the stretch–shortening cycle, improve motor unit recruitment, and increase the rate of force development [10,11]. Furthermore, previous research has indicated that young athletes may be particularly responsive to compound training interventions due to their high adaptive potential during developmental stages [11,12].

Despite the growing body of research investigating the effects of resistance and plyometric training on athletic performance and inter-limb asymmetry, the available evidence remains inconclusive [13,14,15,16]. For example, Falch et al. [17] reported that a six-week training intervention in female handball players produced limited improvements in lower-limb performance and failed to significantly enhance change-of-direction ability. In contrast, Hammami et al. [18] demonstrated that combined upper- and lower-limb plyometric training elicited substantial improvements across multiple physical performance measures in young handball athletes. Similarly, Gonzalo-Skok et al. [19] observed significant reductions in lower-limb asymmetry following a unilateral plyometric training program in soccer players. However, not all studies have reported positive adaptations. Meszler and Vaczi [20], for instance, found no significant changes in agility performance, balance, or the hamstring-to-quadriceps ratio following seven weeks of plyometric training in female basketball players. These inconsistent findings may be attributable to differences in training modality (e.g., plyometric-only versus combined resistance–plyometric protocols), intervention duration (ranging from six to ten weeks across studies), athlete characteristics (age, sex, and competitive level), and the specific outcome measures assessed (e.g., agility, balance, or asymmetry indices). Collectively, these discrepancies underscore a lack of consensus regarding the optimal training approach for reducing lower-limb asymmetry and enhancing performance in team-sport athletes and highlight a specific gap in the literature regarding unilateral compound (combined resistance–plyometric) training protocols evaluated specifically in competitive female handball players.

Importantly, previous research has generally examined unilateral training and compound (combined resistance–plyometric) training as separate approaches rather than evaluating their combined application within a single intervention. Moreover, to the best of our knowledge, no previous study has examined this specific combination, unilateral compound training, in competitive female handball players, a population with neuromuscular and sport-specific demands distinct from those of the cohorts studied previously. Finally, no previous study has simultaneously assessed bilateral strength asymmetry, the hamstring-to-quadriceps (H/Q) ratio, and sport-specific physical performance within the same intervention, despite the likely interrelation of these outcomes. Therefore, the present study was designed to address these gaps by evaluating the effects of a targeted unilateral compound training program on a comprehensive battery of neuromuscular and performance outcomes in competitive female handball players. Based on the aforementioned evidence, the aim of the present study was to examine the effects of a six-week unilateral compound training program on lower-limb strength asymmetry, the hamstring-to-quadriceps ratio, and selected physical performance parameters in competitive young female handball players. We hypothesized that the supplemental unilateral compound training program would be associated with greater reductions in bilateral strength asymmetries and improvements in H/Q ratios than regular handball training alone, together with enhanced sprint, change-of-direction, and jumping performance. Although the intervention was specifically designed around unilateral compound exercises, it is important to acknowledge that the experimental group also completed three additional weekly training sessions compared with the control group. Therefore, the present study was designed to evaluate the effects of a supplemental unilateral compound training program added to regular handball training, rather than to isolate the independent effects of unilateral loading itself. Consequently, any observed benefits should be interpreted as reflecting the combined effects of the unilateral training modality and the additional training volume.

## 2. Materials and Methods

### 2.1. Study Design

A randomized controlled trial with a pre-test–post-test parallel-group design was conducted to examine the effects of a six-week unilateral compound training program on lower-limb strength asymmetry, isokinetic strength, and physical performance in competitive female handball players. Participants were randomly assigned to either an experimental group or a control group and completed identical testing procedures before and after the intervention period. The primary outcomes were bilateral lower-limb strength asymmetry (quadriceps and hamstrings) and hamstring-to-quadriceps (H/Q) ratios. Secondary outcomes included isokinetic strength, sprint performance, change-of-direction ability, countermovement jump performance, single-leg jump performance, and body composition.

### 2.2. Participants

**Figure 1:**
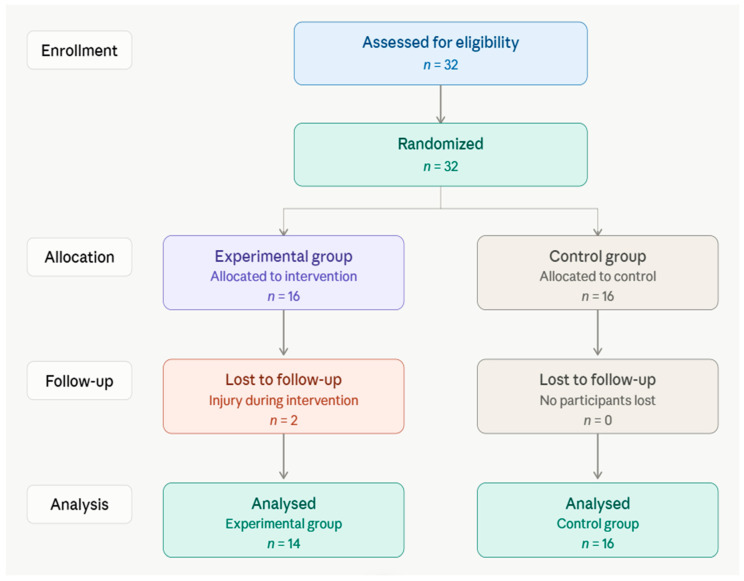
CONSORT Flow Diagram.

Thirty competitive female handball players competing in the Turkish Women’s Second Division voluntarily participated in this study (mean ± SD: age = 18.75 ± 1.91 years, height = 168.98 ± 7.13 cm, body mass = 60.17 ± 7.67 kg). According to the participant classification framework proposed by McKay et al. [21], all athletes were classified as Tier 3 (Highly Trained/National Level). The Turkish Women’s Second Division represents the third competitive tier within the national league structure, below the Super League and First League; nonetheless, participants met McKay et al.’s [21] criteria for Tier 3 classification based on their structured, periodized training under federation-organized competition (four training sessions per week and one official match weekly throughout the season), consistent with the framework’s inclusion of provincial/academy-level competitive structures within this tier rather than top-division status alone. Participants regularly engaged in four handball training sessions per week (approximately 90 min per session) and competed in one official match each weekend throughout the competitive season. These sessions followed the athletes’ standard team training structure, comprising technical skill drills (passing, shooting, dribbling), tactical exercises (offensive and defensive systems, small-sided games), and general conditioning components that inherently involved jumping, sprinting, and change-of-direction movements, without a structured, progressive resistance or plyometric training protocol. An a priori sample size calculation was performed using G*Power (v3.1.9.7) software based on vertical jump and isokinetic strength outcomes reported by Zhang et al. [22]. Specifically, the calculation was based on the effect size reported for the standing long jump (SLJ) outcome in that study (partial η^2^ = 0.31, corresponding to Cohen’s f ≈ 0.67), as this represented the largest and most consistent between-group effect reported among the explosive power measures assessed. According to Cohen’s conventions, this corresponds to a large effect size; although using a large effect size from a related but not identical population (male basketball players) for an a priori calculation carries some risk of underestimating the required sample size, it was considered the most defensible estimate available given the absence of prior data in female handball players undergoing an identical intervention. Assuming an alpha level of 0.05 and a statistical power of 0.95, the analysis indicated that a minimum sample of 26 participants (13 per group) was required (Figure 1). To account for potential dropouts, 32 athletes were initially recruited. The sample size calculation was based on the study by Zhang et al. [22] because it represented the most methodologically comparable study available at the time of study planning. First, the intervention employed a unilateral compound training program that closely matched the present protocol. Second, the primary outcome measures, including vertical jump performance, maximal strength (assessed via isometric mid-thigh pull), and lower-limb asymmetry, were closely aligned with those evaluated in the present study. Third, to the best of our knowledge, no previous study has specifically investigated the effects of unilateral compound training on lower-limb strength asymmetry in competitive female handball players. Although Zhang et al. examined basketball players, both basketball and handball are high-intensity intermittent team sports characterized by comparable neuromuscular demands, including frequent jumping, unilateral force production, sprinting, and rapid changes in direction. Therefore, the reported effect sizes were considered the most appropriate estimates for the a priori sample size calculation. During the intervention period, two participants in the experimental group sustained injuries during their regular handball training sessions that were unrelated to the study intervention, testing procedures, or the unilateral compound training program (one participant sustained a knee injury and the other a hamstring/quadriceps strain). Both injuries were classified as moderate in severity, requiring approximately one to two weeks of absence from training before the athletes were able to return to their regular activities. No injuries were reported or recorded in the control group during the six-week intervention period. Consequently, they withdrew from the study. A complete-case analysis was performed, and only participants who completed both the pre- and post-intervention assessments were included in the final statistical analyses. Therefore, the final sample consisted of 30 athletes (experimental group: *n* = 14; control group: *n* = 16). Following completion of baseline assessments, participants were randomly allocated to either the experimental group or the control group using a computer-generated randomization procedure (https://www.randomizer.org/, accessed on 1 November 2025.). Eligibility criteria included: (i) absence of acute or chronic musculoskeletal injury or disease, as confirmed by the Physical Activity Readiness Questionnaire (PAR-Q); (ii) regular participation in team training at least three times per week; (iii) no history of lower-limb surgery or injury affecting athletic performance; and (iv) no participation in structured resistance or plyometric training during the previous six months.

Compliance with this criterion was verified through a combination of participant self-report and confirmation by the athletes’ team coaches, who were familiar with each player’s individual training history. This verification addressed not only the six-month exclusion window but also participants’ broader training background, as neither the athletes nor their coaches reported any prior exposure, at any point, to structured unilateral resistance or plyometric training. On this basis, none of the participants was identified as having prior experience with structured unilateral resistance or plyometric training, ensuring that all athletes began the intervention with a comparable and naïve training background with respect to the unilateral compound training protocol. Because this assessment relied on self-report and coach recall rather than a standardized, validated training-history instrument, it cannot be entirely excluded that some limited or informal prior exposure went unreported. Future studies should employ structured training-history questionnaires capturing the type, frequency, and duration of prior unilateral training exposure to verify this criterion more objectively. No formal injury-surveillance system was implemented to monitor injury incidence, type, or severity across the full cohort during the intervention period; only the two injuries that led to withdrawal from the study were documented. Consequently, no conclusions can be drawn regarding the protective or risk-modifying effects of the unilateral compound training program on injury occurrence.

All participants received detailed verbal and written information regarding the study objectives, procedures, and potential risks before enrollment. Written informed consent was obtained from all participants and, when applicable, from their legal guardians. Participants were instructed to maintain their habitual dietary practices throughout the intervention period and to refrain from strenuous exercise, alcohol consumption, and stimulant intake for at least 48 h before each testing session. To minimize potential learning effects and improve testing reliability, all athletes completed a one-week familiarization period before baseline assessments. The study was conducted in accordance with the Declaration of Helsinki and was approved by the Mersin University Sports Sciences Ethics Committee, Mersin, Türkiye (Approval No: 080; Date: 20 October 2025).

### 2.3. Experimental Procedures

The study consisted of three sequential phases: (i) baseline assessments, (ii) a six-week intervention period, and (iii) post-intervention assessments. Prior to data collection, all participants completed a one-week familiarization period during which they were introduced to all testing procedures and equipment to minimize potential learning effects and improve measurement reliability.

Baseline testing was conducted over multiple sessions separated by 48 h to minimize the influence of fatigue on performance outcomes. In total, the baseline testing battery spanned approximately eight days (four consecutive 48 h intervals), from the initial body composition assessment to the final isokinetic strength testing session. During this period, participants’ regular competitive match play and high-intensity team training were restricted to prevent residual fatigue from confounding the baseline measurements; only light, low-intensity technical sessions were permitted. This restriction applied equally to both the experimental and control groups, as it pertained specifically to the baseline testing window common to all participants prior to the start of the six-week intervention period; group-specific training (the unilateral compound training program for the experimental group or regular handball training alone for the control group) did not begin until baseline testing was complete. Given the short duration of this restriction (eight days) and the fact that light technical training continued throughout, a meaningful risk of detraining was considered unlikely; detectable declines in strength and power typically require substantially longer periods of reduced training load (generally two weeks or more of near-complete inactivity), and the restriction here involved only a reduction in high-intensity match play and team training rather than complete cessation of physical activity. On the first testing day, body composition was assessed using bioelectrical impedance analysis. Forty-eight hours later, participants completed a standardized warm-up consisting of 5 min of jogging followed by 5 min of dynamic stretching exercises before performing the 20 m sprint test. Forty-eight hours later, lower-limb explosive performance was evaluated using the countermovement jump (CMJ) test on a dual force platform (Hawkin Dynamics, Westbrook, ME, USA). After an additional 48 h recovery period, change-of-direction ability was assessed using the *t*-test. Finally, 48 h later, isokinetic strength testing was performed using a Cybex dynamometer to determine lower-limb peak torque values and calculate bilateral and ipsilateral strength asymmetries. Prior to baseline testing, participants completed a one-week familiarization period during which they attended supervised sessions to practice all testing procedures, including the CMJ, sprint, COD, and isokinetic strength assessments. During these sessions, participants were instructed on the correct execution of each test and were allowed to perform practice trials until they became familiar with the testing procedures. The 10 submaximal repetitions performed immediately before isokinetic testing were intended as a standardized warm-up and final task-specific familiarization rather than the sole familiarization procedure. The familiarization period consisted of three supervised sessions conducted over the week preceding baseline testing. In each session, participants performed three practice trials of each test (CMJ, single-leg CMJ, 20 m sprint, and *t*-test) with real-time feedback on technique from the supervising investigators. For isokinetic testing, participants performed three submaximal and one maximal practice trial at 60°·s^−1^ on each limb. Although this structured familiarization protocol was designed to minimize learning effects, familiarization-session performance data were not formally analyzed to statistically confirm stabilization, and intra- or inter-session reliability coefficients (e.g., intraclass correlation coefficients and coefficients of variation) for the 20 m sprint and change-of-direction tests were not calculated from these sessions; this represents a methodological limitation, discussed further below.

**Figure 2:**
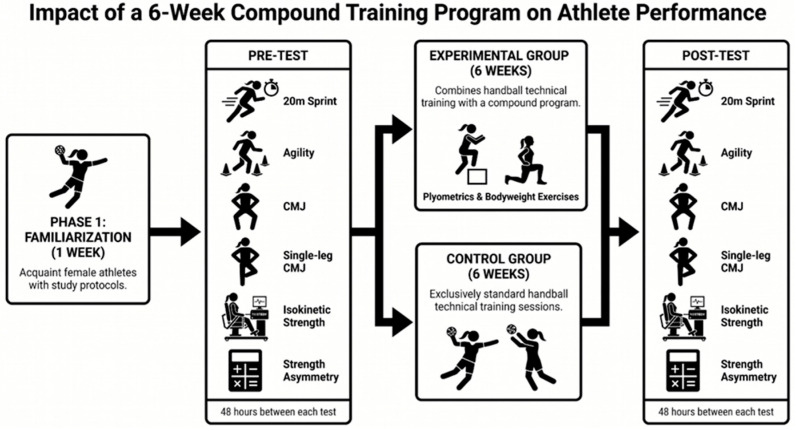
Experimental Design and Testing Timeline.

All assessments were conducted by the same experienced investigators under standardized laboratory conditions and at the same time of day (13:00–16:00 h) to minimize potential circadian influences on performance. These investigators were independent of the coaching staff who delivered the training intervention and were blinded to group allocation during both pre- and post-intervention assessments; participants were also instructed not to disclose their group assignment or discuss training-related details during testing sessions. The same team of investigators conducted both the pre- and post-intervention assessments for all participants, ensuring consistency in testing procedures across time points. Blinding to group allocation was supported by having assessors operate independently of the coaching staff, without access to training logs or attendance records, and by instructing participants not to discuss their training group during testing sessions. Nonetheless, because the experimental and control interventions differed visibly in structure and were delivered by team coaches rather than by the assessment team, complete blinding could not be independently verified. It should also be noted that, given the nature of the intervention, participants themselves could not be blinded to their group assignment; both experimental and control participants were aware of their allocation and of the general nature of the intervention, which may have influenced their motivation, effort, or expectancy during testing and training. Following completion of baseline testing, participants were randomly allocated to either the experimental group (*n* = 14), which performed a six-week unilateral compound training program in addition to regular handball training, or the control group (*n* = 16), which continued regular handball training only. Upon completion of the intervention period, all outcome measures were reassessed using identical procedures and testing conditions. A schematic overview of the study design is presented in Figure 2.

#### 2.3.1. Body Composition Assessment

All measurements were performed in the Physical Profiling, Performance and Biomechanics Laboratory in the Mersin University Faculty of Sport Sciences by the same investigators under standardized testing conditions. Body composition variables, including body mass, body fat percentage, body fat mass, body muscle percentage, and body muscle mass, were assessed using a multi-frequency bioelectrical impedance analyzer (Tanita BC-418, Tanita Corporation, Tokyo, Japan). Participants were instructed to attend testing sessions wearing light sports clothing and barefoot, in accordance with established assessment guidelines [23]. The validity and reliability of the Tanita BC-418 have been previously established, with a strong correlation to hydrostatic weighing (r = 0.81) and excellent same-day test–retest reliability (coefficient of variation = 1.4%; Bland–Altman limits of agreement = 0.91% body fat) reported in a general adult population [23], and acceptable concordance with dual-energy X-ray absorptiometry (ICC = 0.81) reported specifically in an athletic population [24]. Stature was measured to the nearest 0.1 cm using a wall-mounted stadiometer (Holtain Ltd., Crymych, UK), with participants standing barefoot in the Frankfurt plane. Body mass index (BMI) was subsequently calculated as body mass divided by stature squared (kg·m^−2^). To improve measurement consistency, all body composition assessments were performed under standardized laboratory conditions at the same time of day (13:00–16:00 h). Before each testing session, participants were verbally instructed to avoid strenuous exercise, alcohol, and caffeine for at least 24 h and to maintain their habitual dietary practices. Although hydration status, the timing of the last meal, and menstrual cycle phase were not formally standardized or recorded, participants were consistently reminded to attend both baseline and post-intervention assessments under similar daily conditions. Because identical assessment procedures were applied before and after the intervention, any measurement error associated with bioelectrical impedance analysis was expected to affect both assessments similarly.

#### 2.3.2. Countermovement Jump (CMJ) Test

Lower-limb explosive performance was assessed using a dual force platform system (Hawkin Dynamics, USA). Participants performed bilateral countermovement jumps with their hands placed on their hips to eliminate arm swing contribution. From an upright standing position, athletes executed a rapid downward movement followed immediately by a maximal vertical jump. All jumps were performed according to standardized testing procedures and were supervised by the research team. Trials that did not comply with the testing protocol were repeated. Each participant completed three maximal attempts separated by 60 s of passive recovery. The highest jump height achieved was retained for statistical analysis. Jump performance data were automatically recorded and processed using the proprietary Hawkin Dynamics software (https://www.hawkindynamics.com/). Previous research has demonstrated the validity and reliability of the Hawkin Dynamics force platform for assessing vertical jump performance [25].

#### 2.3.3. 20 m Sprint Test

Linear sprint performance was evaluated using an electronic timing system (Fusion Sport SmartSpeed, Brisbane, QLD, Australia). Timing gates were positioned at the start and finish lines of a 20 m sprint course. To standardize the starting procedure, athletes initiated each trial from a standing position located 1 m behind the first timing gate. Participants were instructed to sprint maximally through the finish line without deceleration. Each athlete performed three maximal sprint trials, with adequate recovery provided between attempts. The fastest sprint time was retained for analysis. Because timing was initiated by the photocell beam at the first gate rather than by a starter’s command, reaction time was not included in the recorded sprint time. Photocell-based electronic timing systems, including the SmartSpeed system used in the present study, have been shown to provide valid and reliable measurements of sprint performance in athletic and team-sport populations [26,27].

#### 2.3.4. Change-of-Direction (*t*-Test)

Change-of-direction ability was assessed using the *t*-test according to the protocol described by Pauole et al. [28]. Participants sprinted forward 9.14 m, shuffled 4.57 m to the left, touched the cone, shuffled 9.14 m to the right, touched the opposite cone, shuffled back to the center, and backpedaled to the starting line. Two maximal trials were performed with adequate recovery between attempts, and the fastest time was retained for analysis. The *t*-test has demonstrated high reliability and validity for assessing change-of-direction performance in team-sport athletes [28].

#### 2.3.5. Isokinetic Strength Assessment

Knee extensor and flexor strength were assessed using an isokinetic dynamometer (Humac Norm Cybex II; CSMi Solutions, Stoughton, MA, USA). Prior to testing, participants completed a standardized 10 min warm-up consisting of 5 min of light jogging followed by 5 min of dynamic stretching exercises. To familiarize participants with the testing procedures and minimize potential learning effects, a set of 10 submaximal repetitions was performed at an angular velocity of 180°·s^−1^ before data collection. Following familiarization, concentric knee extension and flexion strength (Con/Con) were evaluated at an angular velocity of 60°·s^−1^ using three sets of five maximal repetitions. Concentric contractions were selected to minimize the risk of hamstring strain during testing [29]. An angular velocity of 60°·s^−1^ was selected because it is the velocity most commonly used to assess maximal isokinetic peak torque and to quantify inter-limb strength asymmetry in the sports science literature, owing to its high measurement reliability and the greater force output typically observed at slower contraction velocities. This velocity also facilitates direct comparison with prior studies examining strength asymmetry in team-sport athletes. These repetitions were performed after completion of the one-week familiarization period and served as a standardized task-specific warm-up and final familiarization immediately before data collection, rather than constituting the entire familiarization procedure. The dynamometer seat was adjusted to 85° according to the manufacturer’s recommendations, and participants were stabilized using trunk and thigh straps to reduce compensatory movements. The ankle was secured to the dynamometer lever arm using a padded cuff, and the range of motion was standardized to 90° for all participants [30]. All assessments were conducted under standardized laboratory conditions between 13:00 and 16:00 h. A 60 s recovery period was provided between sets to minimize fatigue while maintaining neuromuscular activation. Throughout testing, participants received standardized verbal encouragement to ensure maximal effort. Peak torque (PT) values for knee extensors and flexors were recorded in Newton-meters (Nm) and used for subsequent analyses.

#### 2.3.6. Strength Asymmetry Calculations

Bilateral and ipsilateral strength asymmetries were calculated from the peak torque values obtained during the isokinetic assessments. Bilateral asymmetry was determined separately for the quadriceps (Q/Q) and hamstring (H/H) muscles using the following equation:

Bilateral Asymmetry (%) = [(Stronger Limb − Weaker Limb)/Stronger Limb] × 100

This approach provides an estimate of the magnitude of inter-limb strength imbalance independent of limb dominance. This formula was selected because it is the most widely reported method in the strength-asymmetry literature, allowing direct comparison with previous studies employing similar isokinetic protocols [27]. By expressing the deficit relative to the stronger limb rather than to a fixed reference side, this approach avoids conflating asymmetry magnitude with limb dominance and provides an intuitive percentage deficit that is readily interpretable by practitioners. We acknowledge, however, that this formula does not indicate which limb was stronger for a given participant and may over- or underestimate asymmetry magnitude depending on which limb serves as the denominator. Alternative approaches, such as the symmetry angle or a log-transformed ratio, have been proposed to address these limitations by providing a bidirectional and more statistically robust representation of inter-limb differences; these methods were not applied in the present study but represent a valuable direction for future research. Ipsilateral strength balance was assessed using the conventional hamstring-to-quadriceps ratio (H/Q ratio) for both the right and left limbs. The H/Q ratio was calculated according to the equation proposed by Gleim et al. [31]:

H/Q Ratio (%) = (Hamstring Peak Torque/Quadriceps Peak Torque) × 100

The H/Q ratio was used as an indicator of agonist–antagonist muscle balance around the knee joint, with higher values generally reflecting a more favorable balance between knee flexor and extensor muscle groups.

#### 2.3.7. Unilateral Compound Training Intervention

The unilateral compound training program was adapted from the protocol proposed by Zhang et al. [22] and was intended to target lower-limb strength asymmetries while simultaneously enhancing sport-specific performance. Zhang et al. [22] originally examined this approach in male university basketball players over a 10-week period. For the present study, the protocol was adapted to a 6-week duration and applied to competitive female handball players while retaining the core structural elements (exercise selection, asymmetrical loading strategy, and session sequencing) of the original protocol, as these movements are generic lower-limb actions relevant across multiple team sports rather than basketball-specific skills. Specifically, the selected exercises were considered appropriate for handball because they mimic the unilateral demands of handball-specific actions, including single-leg takeoffs during jump shots, unilateral landings following shooting or blocking actions, and single-leg acceleration during rapid directional changes when attacking or defending. These movement patterns are not basketball-specific but instead represent common lower-limb biomechanical demands shared across multiple team sports involving unilateral jumping, landing, and cutting actions, supporting the rationale for adapting the original protocol to a handball-specific context. The intervention was performed three times per week (Monday, Wednesday, and Friday) for six consecutive weeks and was completed immediately after regular handball training sessions. Each session lasted approximately 30–40 min. During the intervention period, the control group continued their regular handball training schedule without any additional strength or plyometric training. The compound training intervention consisted of the sequential combination of unilateral resistance and plyometric exercises performed within the same training session.

Resistance exercises included split squats, Bulgarian split squats, box step-ups, and single-leg calf raises, whereas the plyometric component consisted of lunge jumps, single-leg hops with back-foot raise, single-leg lateral jumps, and single-leg continuous hopping. All exercises were performed using body mass resistance, and participants were instructed to execute each repetition with maximal voluntary effort. Limb dominance was determined a priori via participant self-report (i.e., participants identified their preferred kicking leg) prior to the intervention, rather than being derived from baseline isokinetic strength testing. The designation of the non-dominant limb as the “weaker” limb was therefore based on the common assumption that the non-dominant leg is typically the less trained and comparatively weaker limb in unilaterally dominant sporting actions, rather than on a formal per-participant verification of baseline strength asymmetry direction. To specifically address inter-limb strength deficits, an asymmetrical loading strategy was applied differently across the two training components. For the resistance exercises, each of the four movements was assigned to a single limb: the non-dominant (weaker) limb performed the split squat and box step-up for three sets each, while the dominant limb performed the Bulgarian split squat and single-leg calf raise for one set each. For the plyometric exercises, all four drills were performed bilaterally within the same session, with the non-dominant limb completing three sets of 12 repetitions and the dominant limb completing one set of 12 repetitions for each exercise. This approach was adopted to provide a greater training stimulus to the weaker limb across both training components and thereby facilitate reductions in bilateral strength asymmetry, as previously suggested in unilateral training research [22]. Training volume, exercise order, and load intensity were held constant across the six-week intervention for all participants, consistent with the relatively uniform training-load design of the source protocol [22]; no progressive increase in sets, repetitions, or external load was implemented. This non-progressive design was adopted for two reasons: first, given the relatively short (six-week) duration of the intervention and the need to maintain a consistent training stimulus throughout the competitive season without disrupting handball-specific training demands, a fixed training load was considered appropriate; second, this approach was consistent with the source protocol [22] and avoided introducing progressive overload as an additional variable, thereby simplifying interpretation of the observed adaptations as reflecting the training stimulus applied rather than a confounding increase in load across the intervention period. Rest intervals of 40 s were provided between resistance exercise sets and 60 s between plyometric exercise sets. A 5 min recovery period separated the resistance and plyometric components of each training session. All training sessions were supervised by certified strength and conditioning coaches to ensure exercise compliance, appropriate technique execution, and participant safety. Training attendance was monitored throughout the intervention, and all experimental group participants who completed the study attended 100% of the 18 scheduled training sessions, with no sessions missed. Detailed information regarding the training progression and exercise prescription is presented in Supplementary Table S1.

### 2.4. Statistical Analysis

All statistical analyses were performed using IBM SPSS Statistics (Version 27.0; IBM Corp., Armonk, NY, USA) and GraphPad Prism (Version 10.6.1; GraphPad Software, San Diego, CA, USA). Data are presented as mean ± standard deviation (SD). The normality of data distribution was assessed using the Shapiro–Wilk test, while homogeneity of variances was examined using Levene’s test. Because the within-subject factor (time) had only two levels (pre-test and post-test), the assumption of sphericity was automatically satisfied. Therefore, Mauchly’s test of sphericity and Greenhouse–Geisser corrections were not applicable. Baseline differences between the experimental and control groups were evaluated using independent-samples *t*-tests. To determine the effects of the intervention, a two-way mixed-design ANOVA was performed for all dependent variables, with group (experimental vs. control) treated as the between-subjects factor and time (pre-test vs. post-test) treated as the within-subjects factor. When significant interaction effects were observed, pairwise comparisons were conducted using Bonferroni-adjusted post hoc analyses. Because this study examined multiple outcome domains (body composition, isokinetic strength, physical performance, and asymmetry variables), several mixed-design ANOVAs were conducted. Bonferroni adjustment was applied only to post hoc pairwise comparisons following significant Group × Time interactions. No additional adjustment for multiple comparisons across outcome families (e.g., Bonferroni, Holm–Bonferroni, or false discovery rate) was applied because the study was designed to comprehensively evaluate multiple physiologically related outcomes rather than to test a single predefined primary endpoint. Therefore, the findings should be interpreted with appropriate caution, particularly for variables with borderline statistical significance. It should be noted that a statistically significant Group × Time interaction indicates that the pattern of change over time differed between groups but does not by itself specify the direction of this difference (e.g., greater improvement in the experimental group, a decline in the control group, or both). To clarify the direction and magnitude of change within each group, within-group effect sizes (Cohen’s d) for the pre-to-post change are reported in each results table alongside the interaction statistics. Effect sizes were calculated using partial eta squared (ηp^2^) and interpreted according to the following thresholds: small (ηp^2^ ≥ 0.01), medium (ηp^2^ ≥ 0.06), and large (ηp^2^ ≥ 0.14). Statistical significance was set at *p* < 0.05 for all analyses.

Because two participants, both from the experimental group, withdrew before the post-intervention assessments due to injuries sustained during regular team training unrelated to the intervention, analyses were conducted using a complete-case approach. No missing data imputation methods were applied. An intention-to-treat analysis was not performed. Because both dropouts resulted from injuries sustained during regular team training that were explicitly unrelated to the study intervention, and no valid post-intervention data were available for these participants, a complete-case approach was considered the most appropriate analytical strategy. It should be noted that the non-significant main effect of Group reported for most outcome variables (with the exception of SL-CMJ right and SL-CMJ left, which showed a significant main effect of Group) reflects the average difference between groups collapsed across both pre- and post-intervention time points and should not be interpreted as a direct test of baseline equivalence. Baseline comparability for all primary outcome variables was separately evaluated using independent-samples *t*-tests, as noted above; no significant between-group differences were observed at baseline for any outcome variable (all *p* > 0.05), supporting adequate randomization. Full results of the Shapiro–Wilk and Levene’s tests for all outcome variables are presented in Supplementary Table S2.

## 3. Results

Table 1 presents the baseline characteristics of the participants. No significant differences were observed between the experimental and control groups in age, height, body mass, or body mass index (all *p* > 0.05), indicating successful randomization and baseline comparability on these measured variables prior to the intervention. Although randomization was successful with respect to the anthropometric variables assessed, it does not guarantee equivalence on unmeasured variables (e.g., training history, prior unilateral loading exposure, or baseline neuromuscular status), and the possibility of unmeasured confounding cannot be entirely excluded.

Table 2 presents the two-way mixed-design ANOVA results for body composition variables. Significant Group × Time interaction effects were found for body fat percentage, body muscle percentage, and body muscle mass, indicating that the experimental group demonstrated greater improvements in body composition than the control group following the six-week intervention; no significant interaction effect was observed for body fat mass. The corresponding 95% confidence intervals (0.10–0.56, 0.07–0.53, and 0.27–0.70 for the three significant variables, and 0.00–0.23 for body fat mass) confirmed moderate-to-large intervention effects for the former and a trivial effect for the latter.

Table 3 presents the two-way mixed-design ANOVA results for isokinetic strength variables. Significant Group × Time interaction effects were found for right knee flexion, left knee extension, and left knee flexion, whereas no significant interaction effect was observed for right knee extension.

**Figure 3:**
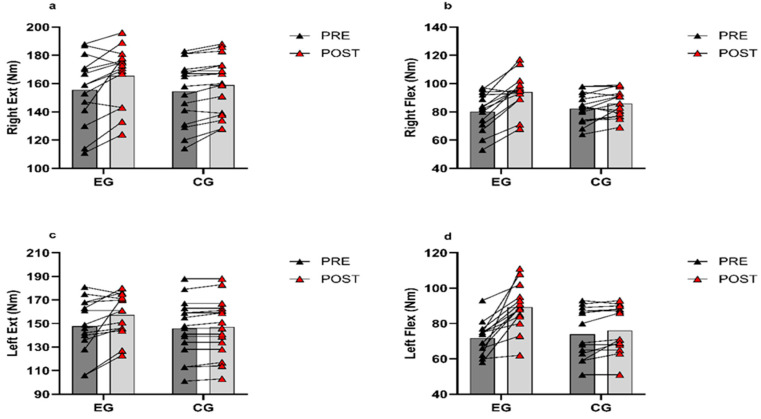
Pre- and post-intervention isokinetic strength values for the experimental group (EG) and control group (CG). (**a**) Right knee extension (Right Ext, Nm); (**b**) Right knee flexion (Right Flex, Nm); (**c**) Left knee extension (Left Ext, Nm); (**d**) Left knee flexion (Left Flex, Nm). Black triangles represent pre-test values; red triangles represent post-test values.

The corresponding 95% confidence intervals for these interaction effect sizes (right knee extension: 0.00–0.33; right knee flexion: 0.08–0.54; left knee extension: 0.01–0.42; left knee flexion: 0.16–0.62) indicated moderate-to-large intervention effects for knee flexor strength and a small-to-moderate effect for left knee extensor strength, whereas the effect for right knee extensor strength was comparatively smaller and did not reach statistical significance. Descriptively, right-limb peak torque values exceeded left-limb values for both quadriceps and hamstring muscle groups across both groups and both testing sessions, indicating that the right limb was consistently the stronger limb in the present sample. This directional pattern was considered when interpreting the bilateral asymmetry percentages reported below, as the “stronger limb” denominator in the asymmetry formula corresponded to the right limb for the majority of participants. The lack of statistical significance for right knee extension may partly reflect its comparatively higher baseline (pre-test) strength relative to the left limb across both groups, potentially leaving less room for a detectable training-induced improvement; the relationship between this side-specific pattern and limb dominance is examined in detail, including important methodological caveats, in Section 4. The pre- and post-intervention isokinetic strength values for both groups are presented in Figure 3.

**Figure 4:**
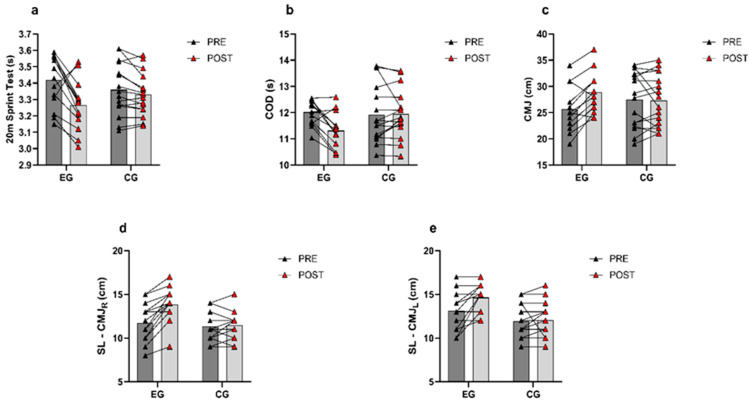
Pre- and post-intervention physical performance values for the experimental group (EG) and control group (CG). (**a**) 20 m sprint test (s); (**b**) Change-in-direction test (COD, s); (**c**) Countermovement jump (CMJ, cm); (**d**) Single-leg CMJ right (SL-CMJ_R, cm); (**e**) Single-leg CMJ left (SL-CMJ_L, cm). Black triangles represent pre-test values; red triangles represent post-test values.

Table 4 presents the two-way mixed-design ANOVA results for physical performance variables. Unlike the other variables, significant group main effects were found for SL-CMJ right and SL-CMJ left. Significant Group × Time interaction effects were observed for all variables, indicating that the experimental group demonstrated significantly greater improvements across all physical performance measures compared to the control group. The corresponding 95% confidence intervals for these interaction effect sizes (20 m sprint: 0.02–0.46; COD: 0.07–0.54; CMJ: 0.06–0.52; SL-CMJ right: 0.18–0.63; SL-CMJ left: 0.01–0.42) supported moderate-to-large intervention effects across all outcomes, with the largest effect observed for SL-CMJ right. Inspection of within-group effect sizes (Table 4) indicated large pre-to-post changes in the experimental group (d = 0.80–1.22) alongside negligible-to-small changes in the control group (d = 0.03–0.21), supporting the interpretation that the observed interactions reflected a substantially greater training response in the experimental group rather than a decline in the control group. The pre- and post-intervention physical performance values are presented in Figure 4.

Table 5 presents the two-way mixed-design ANOVA results for lower-extremity asymmetry variables. Significant Group × Time interaction effects were found for all variables, indicating that the experimental group demonstrated significant reductions in lower-limb strength asymmetry and improvements in hamstring-to-quadriceps ratios compared with the control group. The corresponding 95% confidence intervals for these interaction effect sizes (bilateral quadriceps asymmetry: 0.06–0.52; bilateral hamstring asymmetry: 0.10–0.57; right H/Q ratio: 0.02–0.44; left H/Q ratio: 0.05–0.50) supported moderate-to-large intervention effects, indicating that the unilateral compound training program effectively reduced bilateral lower-limb strength asymmetry and improved hamstring-to-quadriceps ratios compared with regular handball training.

**Figure 5:**
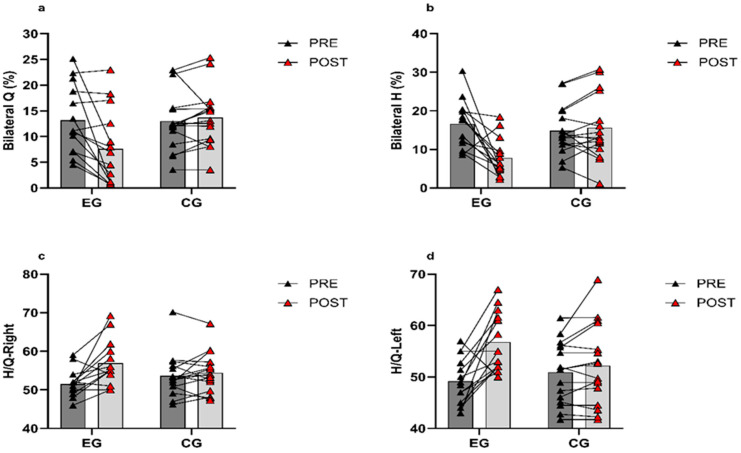
Pre- and post-intervention lower-extremity asymmetry values for the experimental group (EG) and control group (CG). (**a**) Bilateral quadriceps asymmetry (Bilateral Q, %); (**b**) Bilateral hamstring asymmetry (Bilateral H, %); (**c**) Hamstring-to-quadriceps ratio for the right limb (H/Q-Right, %); (**d**) Hamstring-to-quadriceps ratio for the left limb (H/Q-Left, %). Black triangles represent pre-test values; red triangles represent post-test values.

Post hoc power analysis demonstrated excellent statistical power for the primary and principal performance outcomes (achieved power exceeded 0.99 in all cases), confirming that the final sample size of 30 participants was sufficient to detect the observed intervention effects. This post hoc power analysis is reported for descriptive completeness only; because it is calculated directly from the observed effect sizes, it is mathematically circular for statistically significant outcomes and should not be interpreted as independent confirmation of an adequate sample size. The primary justification for the study’s sample size is the a priori power analysis reported in Section 2, which was conducted using G*Power prior to data collection based on effect sizes from comparable prior literature [22] and indicated that a minimum of 26 participants (13 per group) was required. A total of 32 athletes were recruited, and 30 completed the study after two injury-related withdrawals (see Figure 5).

## 4. Discussion

The primary finding of the present study was the significant reduction in bilateral quadriceps and hamstring strength asymmetries following the six-week unilateral compound training intervention. In parallel, both right and left hamstring-to-quadriceps (H/Q) ratios improved significantly in the experimental group, indicating a more balanced force-generating capacity between agonist and antagonist muscle groups around the knee joint. These findings suggest that supplemental unilateral compound training may represent a promising strategy for addressing inter-limb strength deficits while simultaneously improving knee joint muscle balance in female handball players.

It should be acknowledged that the control group’s regular handball training, although lacking a structured resistance or plyometric protocol, inherently incorporated jumping, sprinting, and change-of-direction actions through technical drills, small-sided games, and competitive match play. Consistent with this, the control group exhibited small but measurable improvements in several performance and strength variables (e.g., a 0.89% improvement in 20 m sprint time, compared with 4.68% in the experimental group). Such modest gains are expected in athletes engaged in regular sport-specific training and likely reflect the natural training stimulus embedded within team practice and competition, rather than a targeted overload effect. These control group findings underscore the value of the parallel-group design: without a comparison group, the improvements observed in the experimental group could not be confidently distinguished from seasonal training effects or residual familiarization. The substantially larger improvements observed in the experimental group relative to the control group therefore provide supportive evidence that the unilateral compound training program conferred a benefit beyond that offered by standard team training alone, particularly for reducing inter-limb strength asymmetry, an outcome not directly targeted by standard team training; as noted elsewhere; however, this benefit cannot be fully attributed to the unilateral training modality specifically, as opposed to the additional training volume.

The observed reductions in bilateral quadriceps and hamstring asymmetries, together with the improvements in H/Q ratios, indicate that the supplemental unilateral compound training program was associated with enhanced lower-limb muscle balance in the present cohort of female handball players. Handball is characterized by frequent unilateral actions such as jumping, landing, cutting, throwing, and accelerating, which may promote preferential loading of one limb and contribute to the development of both inter-limb and agonist–antagonist asymmetries over time [3]. Consequently, interventions capable of restoring lower-limb strength symmetry may provide important benefits for both performance enhancement and injury prevention. In this regard, it should be emphasized that the present study did not directly assess injury outcomes and therefore cannot provide evidence regarding the injury prevention benefits of this training approach; any such benefit remains hypothetical, and the injury prevention implications noted here are drawn from the broader literature rather than from the present findings. These findings are consistent with previous research reporting substantial strength imbalances in female handball athletes. Xaverova et al. [32] identified pronounced bilateral deficits in knee flexor and extensor strength, accompanied by relatively low H/Q ratios, and highlighted the importance of targeted strengthening programs, while Barreto et al. [33] similarly demonstrated significant between-limb differences in knee extensor strength and H/Q ratios, suggesting that these imbalances are modifiable through systematic training. Our findings also extend evidence from other unilateral training interventions: Zhang et al. [22] demonstrated that a unilateral compound training program significantly reduced lower-limb asymmetry in male basketball players, and Gonzalo-Skok et al. [19] reported comparable improvements following unilateral plyometric training in soccer players. A plausible mechanistic explanation for the present improvements is the asymmetrical loading strategy incorporated within the training program: by allocating a greater training volume to the non-dominant (weaker) limb, the intervention likely promoted greater neuromuscular adaptations in the weaker extremity, thereby reducing inter-limb discrepancies. It should first be noted that Table 3 reports isokinetic strength values by anatomical side (right/left) rather than by limb dominance, whereas limb dominance was individually self-reported and was mixed across participants (i.e., not uniformly right- or left-dominant). Therefore, the side-specific pattern observed in Table 3 (greater improvement in the left limb, and a non-significant interaction for right knee extension) cannot be mechanistically attributed to the dominance-based asymmetrical loading strategy, since anatomical side did not consistently correspond to limb dominance across participants. In contrast, the asymmetry analyses presented in Table 5 were calculated on a dominance-specific basis (dominant vs. non-dominant) for each participant and therefore more directly reflect the intended effect of the asymmetrical training allocation, namely, that allocating greater training volume to the non-dominant limb promoted greater neuromuscular adaptation in the weaker extremity and reduced inter-limb strength asymmetry. Future studies should consider reporting raw strength values by dominance status rather than anatomical side, to allow more direct mechanistic interpretation of limb-specific training effects. This interpretation is supported by Zhang et al. [22], who demonstrated that unilateral compound training significantly reduced lower-limb asymmetry in male basketball players and that these reductions were associated with improvements in explosive performance and maximal strength. Collectively, these findings suggest that supplemental unilateral compound training may represent a promising and practical approach for restoring lower-limb strength balance in female handball players while simultaneously creating a more favorable neuromuscular environment for performance development. While these mechanisms are plausible based on previous research, the present study did not directly assess neuromuscular adaptations (e.g., via electromyography) or muscle architecture (e.g., via ultrasound), and therefore these explanations should be considered speculative. These reductions should be interpreted with appropriate caution given their magnitude. Importantly, post-intervention asymmetry values for both quadriceps (7.60%) and hamstring (7.83%) fell below the 10% threshold commonly associated with elevated injury risk, suggesting that these changes may be clinically meaningful rather than merely statistical artifacts, although, as noted above, the present study did not directly assess injury outcomes and cannot confirm whether this reduction translated into an actual decrease in injury risk. It should also be noted that this 10% threshold is derived from studies that have variably employed the percentage-difference method, the symmetry angle, or other asymmetry indices, and comparisons across studies using different calculation approaches should therefore be interpreted with caution. The magnitude of these reductions is also consistent with, and in some cases smaller than, reductions reported in the source protocol study [22], in which comparable asymmetry measures decreased by 48.5% and 64.7%. Regression to the mean cannot be entirely excluded as a contributing factor, particularly given the considerable inter-individual variability in baseline asymmetry (SD ≈ 6–7%). However, because both groups were randomly drawn from the same population and displayed comparable baseline asymmetry values and variability, regression to the mean would be expected to affect the control group to a similar extent. The fact that the control group showed no corresponding reduction, despite comparable baseline asymmetry, therefore argues against regression to the mean as the primary explanation for the changes observed in the experimental group.

Reducing lower-limb strength asymmetry is theoretically important for optimizing athletic performance, although the present study did not directly assess injury outcomes and therefore cannot draw conclusions regarding injury prevention. Inter-limb strength imbalances may impair force production, movement efficiency, and the execution of sport-specific tasks requiring high levels of power and neuromuscular coordination. Likewise, asymmetries have been associated with reduced sprint, change-of-direction performance, dynamic balance, and movement control [34]. Although Lockie et al. [35] reported that moderate asymmetries may have limited influence on sprint and agility performance, larger asymmetries were associated with poorer athletic performance. Accordingly, the reductions in bilateral strength asymmetry observed in the present study may have contributed to the concurrent improvements in sprint, change-of-direction, and jumping performance, potentially through more efficient force production and reduced compensatory movement strategies.

The present study demonstrated significant improvements in sprint speed, change-of-direction ability, and both bilateral and unilateral jumping performance following the six-week supplemental unilateral compound training intervention. These findings indicate that combining unilateral resistance and plyometric exercises as a supplemental training block may provide a beneficial neuromuscular stimulus for enhancing performance capacities that are essential in handball, including acceleration, rapid directional changes, and explosive jumping. Our findings are consistent with previous studies reporting improvements in sprint, change-of-direction, and jumping performance following combined strength and plyometric training in female handball players [18,36], as well as plyometric [37] and combined isometric–plyometric interventions [38]. Collectively, the available evidence suggests that training strategies integrating strength- and power-oriented stimuli are effective for improving sport-specific physical performance in female handball athletes.

The improvements in sprint performance may be explained by the inclusion of unilateral plyometric exercises emphasizing horizontal force production. Sprint acceleration is strongly associated with the ability to generate horizontal propulsive forces [39,40], and exercises such as single-leg hops and lunge jumps may have enhanced the athletes’ capacity to apply force more effectively in the horizontal direction. Moreover, the biomechanical specificity of these exercises to sprinting likely facilitated transfer to sprint performance [41]. The observed 4.68% improvement exceeded the smallest worthwhile change (SWC) of approximately 1% for 20 m sprint performance in team-sport athletes [26], suggesting that the improvement was not only statistically significant but also practically meaningful. Although the parallel-group design supports the interpretation that these gains primarily reflect the training intervention, the possibility that the small improvement observed in the control group was partially influenced by residual learning effects beyond the one-week familiarization period cannot be completely excluded. Similarly, improvements in change-of-direction performance are likely attributable to neuromuscular adaptations induced by the combined resistance and plyometric training, including enhanced motor unit recruitment, neural drive, and intermuscular coordination [42]. Although the relative contribution of strength and plyometric training to change-of-direction performance remains debated [43,44], the present findings support the view that combining both training modalities provides complementary adaptations that enhance directional-change ability. It should also be noted that the *t*-test assesses pre-planned change-of-direction performance rather than reactive agility and therefore may not fully reflect the perceptual and decision-making demands of competitive handball. Future studies should incorporate sport-specific reactive agility tests to further evaluate the transfer of unilateral compound training to match performance.

The improvements observed in both bilateral and unilateral jumping performance are consistent with previous studies demonstrating that plyometric and strength-oriented training enhance jumping ability in female athletes [20,45,46]. The present findings extend this evidence by suggesting that supplemental unilateral compound training is associated with improvements in both bilateral and single-leg jump performance, which is particularly relevant to handball given the unilateral demands of many sport-specific actions. These improvements may be explained by enhanced muscle–tendon stiffness, more effective utilization of elastic energy during the stretch–shortening cycle, and neuromuscular adaptations, including increased motor unit recruitment and intermuscular coordination [47]. Furthermore, the concurrent reduction in lower-limb asymmetry may have promoted a more balanced distribution of force between limbs, thereby contributing to the observed improvements in jumping performance. As with the mechanisms discussed above, these neuromuscular and biomechanical explanations remain speculative in the absence of direct electromyographic or muscle architecture assessments in the present study. The reduction in body fat should also be interpreted with some caution. Although the observed 4.05-percentage-point decrease exceeded the reported test–retest measurement error of the BIA device (coefficient of variation = 1.4%), hydration status, timing of the last meal, and menstrual cycle phase were not standardized and may have influenced the estimates. Consequently, these findings should not be interpreted as unequivocal evidence of physiological adaptation. Nevertheless, if confirmed using more precise body-composition techniques, a reduction in this magnitude could be considered practically beneficial because a lower body fat percentage is generally associated with an improved power-to-mass ratio, which may contribute to sprint, jumping, and change-of-direction performance.

A central methodological consideration for interpreting the present findings is the inability to disentangle the effects of the unilateral training modality from those of the additional training volume. The experimental group completed three additional weekly sessions beyond regular handball training, while the control group received no additional training; consequently, the observed improvements could reflect the specific unilateral loading pattern, the simple addition of training volume, or an interaction of both factors. This is a common limitation in supplemental-training designs and cannot be resolved with the present data. A volume-matched active control, for example, a bilateral compound resistance–plyometric program of equal frequency and duration, would be required to isolate the contribution of the unilateral loading pattern specifically. In the absence of such a comparison, the present findings should be interpreted as demonstrating the benefit of adding this supplemental unilateral compound training block to regular handball training, rather than establishing the superiority of unilateral loading over an equivalent bilateral alternative.

Several limitations of the present study should be acknowledged. First, the findings are limited to competitive young female handball players competing in the Turkish Women’s Second Division over a six-week intervention period and therefore may not be generalizable to male athletes, other age groups, athletes from other sports, or players competing at different competitive levels; in particular, elite athletes may respond differently to this training stimulus than the sub-elite population studied here, given differences in baseline strength, training history, and neuromuscular status. Although the final sample size (*n* = 30) exceeded the minimum estimated by the a priori power analysis, the relatively modest sample may have reduced the ability to detect smaller intervention effects. Complete blinding could not be independently verified: because the experimental and control interventions differed visibly in structure and were delivered by team coaches rather than by the assessment team, assessors could plausibly have inferred group allocation despite being nominally blinded, and the possibility that assessor expectations influenced the recorded outcomes cannot be excluded; double-blinding is inherently difficult to achieve in exercise intervention research of this kind, and single-blinding of outcome assessors was therefore only partially successful. Furthermore, because the experimental group completed three additional weekly unilateral compound training sessions, the observed benefits cannot be attributed exclusively to the training modality itself rather than the greater overall training volume. The intervention was also limited to six weeks, and only immediate post-intervention outcomes were assessed; no follow-up testing was conducted to determine whether the observed improvements were retained over time. No formal injury-surveillance system was implemented beyond documenting the two participant withdrawals, and because these two participants withdrew before study completion, a complete-case rather than intention-to-treat analysis was performed, such that the possibility of missing-data bias cannot be entirely excluded.

With respect to measurement, no direct neuromuscular measurements (e.g., electromyography, muscle architecture, or biomechanical analyses) were obtained. Although training attendance was monitored and reached 100%, the quality, intensity, and technical execution of individual training sessions were not objectively assessed via heart rate monitoring, session ratings of perceived exertion (sRPE), or movement quality evaluation; consequently, potential variability in the actual training stimulus and technical execution experienced by individual athletes cannot be ruled out. Future studies should incorporate objective load-monitoring tools such as sRPE or heart rate monitoring, together with movement quality assessments. Isokinetic strength was assessed only at a single angular velocity (60°·s^−1^) under concentric conditions, and may therefore not fully capture high-velocity force production, rate of force development, or eccentric strength capacities relevant to handball performance; the present findings may not generalize to other movement velocities, and future studies should test at multiple angular velocities (e.g., 60°, 180°, and 240°·s^−1^) and include eccentric assessments. Bilateral asymmetry was calculated using the percentage-difference method based on the stronger limb; although widely used, this approach does not provide information regarding asymmetry direction, may be influenced by the selected denominator, and, as a ratio-based measure, may not always satisfy the assumptions of parametric statistical analyses. Alternative approaches, including the symmetry angle and log-transformed ratio, should be considered in future studies. Body composition was assessed using bioelectrical impedance analysis, which may be influenced by hydration status, glycogen availability, recent dietary intake, and menstrual cycle phase despite standardized testing procedures; future studies should consider dual-energy X-ray absorptiometry (DXA) or air displacement plethysmography (Bod Pod) for more precise assessment. Although participants completed a structured one-week familiarization period, familiarization-session data were not formally analyzed to confirm performance stabilization; consequently, learning effects, particularly for the 20 m sprint and change-of-direction tests, cannot be completely excluded and may partly explain the small improvements observed in the control group. Future studies should report familiarization reliability statistics (e.g., ICC and coefficient of variation).

From a statistical perspective, the present study involved a large number of dependent variables. Although Bonferroni-adjusted post hoc comparisons were performed following significant interaction effects, no global correction for multiple testing across outcome families was applied, and the possibility of inflated Type I error cannot be excluded, particularly for variables with marginal *p*-values. The findings should therefore be interpreted as exploratory and hypothesis-generating rather than strictly confirmatory, and future confirmatory studies with predefined primary outcomes and appropriate multiplicity adjustment are warranted. A post hoc power analysis indicated excellent statistical power (achieved power > 0.99) for the primary outcomes; however, because this calculation is derived directly from the observed effect sizes, it is mathematically circular for statistically significant results and should not be interpreted as independent confirmation of an adequate sample size, the a priori power analysis reported in the Methods remains the primary justification for the study’s sample size.

Despite these limitations, the study has several important strengths, including its randomized controlled design, the use of isokinetic dynamometry, and the simultaneous evaluation of bilateral strength asymmetry, H/Q ratios, and sport-specific performance outcomes. Moreover, the unilateral compound training program required only minimal equipment and approximately 30 min per session, three times per week, supporting its practical feasibility for team-sport settings, although qualified supervision remains important; nonetheless, coaches implementing this program should be aware that, in the absence of objective load monitoring, the individual training stimulus may vary and should ideally be tracked (e.g., via sRPE) in practice. Future research should investigate the long-term effects of this training approach across different competitive levels, sexes, and age groups while incorporating neuromuscular, biomechanical, and injury-related outcomes.

## 5. Conclusions

The present study demonstrated that a six-week supplemental unilateral compound training program, added to regular handball training, was associated with reduced lower-limb strength asymmetries and simultaneous improvements in isokinetic strength, sprint performance, change-of-direction ability, and both bilateral and unilateral jumping performance in competitive female handball players. Because the experimental group performed three additional weekly sessions relative to the control group, these improvements may be attributable to the specific unilateral training modality, the additional training volume, or a combination of both, and should not be interpreted as demonstrating the specific superiority of unilateral training per se. These findings suggest that combining unilateral resistance and plyometric exercises as a supplemental training block may represent a potentially promising strategy that warrants further investigation for enhancing neuromuscular performance and restoring lower-limb strength balance in athletes exposed to repeated unilateral sport-specific demands. From a practical perspective, coaches and practitioners may consider implementing supplemental unilateral compound training as an addition to regular handball training; the program can be completed in approximately 30 min, three times per week, with minimal equipment, supporting its feasibility for team-sport settings. This approach may be particularly relevant for athletes presenting with pronounced inter-limb strength asymmetries, such as those whose weaker limb is more than 10% below the stronger limb, a threshold commonly associated with elevated injury risk in the broader literature, though derived from studies using varying asymmetry calculation methods and therefore not directly equivalent across all metrics, providing coaches and practitioners with a practical method for simultaneously addressing inter-limb strength deficits and improving key performance attributes relevant to handball. Based on associations reported in the broader literature, rather than on findings from the present study, between strength asymmetry and injury risk, regular monitoring of lower-limb asymmetries and individualized corrective training may be a reasonable practical consideration for female handball athletes. We emphasize that the present study did not directly assess injury outcomes and therefore cannot provide evidence regarding the injury prevention benefits of this training approach; any such benefit remains hypothetical and should be confirmed by future research incorporating injury-surveillance data. Importantly, because the present design cannot disentangle the effects of the unilateral training modality from those of the additional training volume, future studies should employ a volume-matched active control condition, such as a bilateral compound resistance–plyometric program of equal frequency and duration, to clarify whether the benefits observed here are specific to unilateral loading or are attributable more generally to the added training stimulus. Future studies should also investigate the long-term effects of supplemental unilateral compound training across different age groups, competitive levels, and intervention durations. Additionally, research incorporating biomechanical, neuromuscular, and injury-related outcomes may further clarify the mechanisms through which asymmetry reduction contributes to performance enhancement and injury prevention. These findings should be considered exploratory and require confirmation in adequately powered confirmatory trials using predefined primary outcomes and appropriate adjustment for multiple comparisons. Given the observed relationship between baseline asymmetry and intervention response, coaches should consider monitoring individual athletes’ asymmetry profiles when deciding whether to implement this program, as athletes with minimal baseline asymmetry may derive comparatively smaller benefits from this training approach and could instead prioritize other performance-oriented training modalities.

## Figures and Tables

**Table 1:** Baseline Characteristics of the Participants.

	Experimental Group (n: 14) (X^−^ ± SD)	Control Group (n: 16) (X^−^ ± SD)	t	*p*
Age (Years)	19.07 ± 2.06	18.44 ± 1.75	0.91	0.37
Height (cm)	168.07 ± 6.98	169.88 ± 7.39	−0.68	0.50
Body Mass (kg)	60.98 ± 9.39	59.36 ± 5.54	0.58	0.57
BMI (kg·m^−2^)	21.57 ± 2.96	20.56 ± 1.30	1.18	0.25

Data are presented as mean ± SD.

**Table 2:** Two-Way Mixed-Design ANOVA Results for Body Composition Variables.

Variable	Group	Pre-Test (X^−^ ± SD)	Post-Test (X^−^ ± SD)	%Δ	d	*p*-Value (ηp^2^)					
						Time (*p*)	ηp^2^	Group (*p*)	ηp^2^	Interaction (*p*)	ηp^2^
Body Fat (%)	EG	23.93 ± 5.79	19.88 ± 4.05	−16.92	0.81	0.001	0.74	0.232	0.05	0.001	0.37
	CG	24.33 ± 2.72	22.83 ± 2.04	−6.17	0.62						
Body Fat (kg)	EG	15.15 ± 5.86	13.14 ± 4.38	−13.27	0.39	0.001	0.47	0.473	0.02	0.319	0.04
	CG	15.79 ± 1.90	14.47 ± 2.14	−8.36	0.65						
Body Muscle (%)	EG	74.00 ± 5.79	76.82 ± 3.19	3.81	0.60	0.008	0.23	0.475	0.02	0.001	0.32
	CG	74.37 ± 4.63	74.02 ± 4.97	−0.47	0.07						
Body Muscle (kg)	EG	43.99 ± 5.40	46.96 ± 5.28	6.75	0.56	0.001	0.77	0.594	0.01	0.001	0.55
	CG	44.18 ± 4.35	44.89 ± 4.24	1.61	0.17						

Note: Time (*p*) and Group (*p*) represent the main effects of time and group, respectively, whereas Interaction (*p*) represents the Group × Time interaction effect derived from the two-way mixed-design ANOVA. EG = Experimental Group; CG = Control Group; ηp^2^ = partial eta-squared.

**Table 3:** Two-Way Mixed-Design ANOVA Results for Isokinetic Strength Variables.

Variable	Group	Pre Test (X^−^ ± SD)	Post Test (X^−^ ± SD)	%Δ	d	*p*-Value (ηp^2^)					
						Time (*p*)	ηp^2^	Group (*p*)	ηp^2^	Interaction (*p*)	ηp^2^
Right Ext (Nm)	EG	155.57 ± 24.73	165.57 ± 21.43	6.43	0.43	0.001	0.44	0.638	0.01	0.072	0.11
	CG	154.63 ± 22.20	153.94 ± 20.36	−0.45	0.03						
Right Flex (Nm)	EG	80.14 ± 13.61	94.00 ± 13.27	17.29	1.03	0.001	0.602	0.470	0.02	0.001	0.34
	CG	82.31 ± 10.18	85.94 ± 9.17	4.41	0.37						
Left Ext (Nm)	EG	147.79 ± 23.51	157.36 ± 18.27	6.48	0.45	0.002	0.29	0.459	0.02	0.015	0.20
	CG	145.81 ± 24.15	147.06 ± 24.07	0.86	0.05						
Left Flex (Nm)	EG	71.86 ± 9.23	89.29 ± 12.94	24.26	1.55	0.001	0.56	0.193	0.06	0.001	0.44
	CG	73.94 ± 13.43	76.06 ± 12.71	2.88	0.16						

Note: Time (*p*) and Group (*p*) represent the main effects of time and group, respectively, whereas Interaction (*p*) represents the Group × Time interaction effect. *p* < 0.05; EG = Experimental Group; CG = Control Group; ηp^2^ = partial eta squared; Ext = Extension; Flex = Flexion; Nm = Newton-meter.

**Table 4:** Two-Way Mixed-Design ANOVA Results for Physical Performance Variables.

Variable	Group	Pre Test (X^−^ ± SD)	Post Test (X^−^ ± SD)	%Δ	d	*p*-Value (ηp^2^)					
						Time (*p*)	ηp^2^	Group (*p*)	ηp^2^	Interaction (*p*)	ηp^2^
20 m Sprint (s)	EG	3.42 ± 0.16	3.26 ± 0.15	−4.68	1.03	0.001	0.41	0.949	0.01	0.006	0.24
	CG	3.36 ± 0.16	3.33 ± 0.13	−0.89	0.21						
COD (s)	EG	12.02 ± 0.48	11.31 ± 0.67	−5.91	1.22	0.002	0.29	0.363	0.03	0.001	0.33
	CG	11.92 ± 1.11	11.95 ± 0.93	0.25	0.03						
CMJ (cm)	EG	25.64 ± 4.14	28.86 ± 3.74	12.56	0.82	0.003	0.27	0.929	0.01	0.001	0.32
	CG	27.48 ± 5.33	27.31 ± 4.94	−0.62	0.03						
SL-CMJ_R_ (cm)	EG	11.71 ± 2.20	13.86 ± 1.92	18.36	1.04	0.001	0.52	0.040	0.14	0.001	0.46
	CG	11.31 ± 1.82	11.44 ± 1.59	1.15	0.08						
SL-CMJ_L_ (cm)	EG	13.14 ± 2.14	14.64 ± 1.55	11.42	0.80	0.005	0.25	0.006	0.23	0.015	0.19
	CG	11.94 ± 1.88	12.06 ± 1.95	1.01	0.06						

Note: Time (*p*) and Group (*p*) represent the main effects of time and group, respectively, whereas Interaction (*p*) represents the Group × Time interaction effect. *p* < 0.05; EG = Experimental Group; CG = Control Group; ηp^2^ = partial eta squared; s = seconds; cm = centimeter; COD = Change-in-Direction; CMJ = Countermovement Jump; SL-CMJR = Single-Leg Countermovement Jump (Right); SL-CMJL = Single-Leg Countermovement Jump (Left).

**Table 5:** Two-Way Mixed-Design ANOVA Results for Lower-Extremity Asymmetry Variables.

Variable	Group	Pre Test (X^−^ ± SD)	Post Test (X^−^ ± SD)	%Δ	d	*p*-Value (ηp^2^)					
						Time (*p*)	ηp^2^	Group (*p*)	ηp^2^	Interaction (*p*)	ηp^2^
Bilateral Q (%)	EG	13.19 ± 6.62	7.60 ± 7.47	−42.38	0.79	0.011	0.21	0.181	0.06	0.002	0.31
	CG	13.00 ± 5.76	13.69 ± 5.63	5.31	0.12						
Bilateral H (%)	EG	16.65 ± 6.23	7.83 ± 5.01	−52.97	1.56	0.002	0.30	0.171	0.07	0.001	0.37
	CG	14.86 ± 6.26	15.60 ± 8.41	4.98	0.10						
H/Q–Right (%)	EG	51.53 ± 3.62	56.95 ± 5.84	10.52	1.12	0.001	0.34	0.904	0.01	0.009	0.22
	CG	53.64 ± 5.51	54.43 ± 5.21	1.66	0.15						
H/Q–Left (%)	EG	49.18 ± 4.53	56.82 ± 5.65	15.53	1.49	0.001	0.45	0.493	0.02	0.002	0.29
	CG	50.92 ± 5.97	52.22 ± 7.83	2.55	0.19						

Note: Time (*p*) and Group (*p*) represent the main effects of time and group, respectively, whereas Interaction (*p*) represents the Group × Time interaction effect. *p* < 0.05; EG = Experimental Group; CG = Control Group; ηp^2^ = partial eta squared; Q = Quadriceps; H = Hamstring; H/Q = Hamstring-to-Quadriceps ratio.

## Data Availability

The data presented in this study are available on request from the corresponding author. The data are not publicly available due to privacy restrictions.

## Supplementary Materials

The following supporting information can be downloaded at: https://www.mdpi.com/article/10.3390/life16071169/s1, Table S1: Detailed Exercise Prescription for the Six-Week Unilateral Compound Training Program; Table S2: Results of the Shapiro-Wilk and Levene Tests.
