# Supplementary material for: Unilateral Compound Training Reduces Lower-Limb Strength Asymmetry and Enhances Athletic Performance in Female Handball Players: A Randomized Controlled Trial
Source: Life (Basel). 2026 Jul 15;16(7):1169. doi: 10.3390/life16071169 (PMC13413411; doi:10.3390/life16071169)
Supplement: Supplementary file 1 [file life-16-01169-s001.zip › life-4406419-supplementary.pdf]

Table S1. Detailed Exercise Prescription for the Six-Week Unilateral Compound Training Program

Group	Training Component	Exercise	Description / Execution Technique	Target Limb	Volume (Sets × Reps)	Rest Interval	Block Duration (min)	Training Intensity
EG	Resistance	1. Split Squat	Staggered split stance; front (non-dominant) knee flexes to ~90° while torso remains upright, then returns to start.	Non-dominant	3 × 6	40 s	13	Bodyweight resistance; maximal voluntary effort under coach supervision
		2. Bulgarian Split Squat	Rear-foot-elevated split squat; dominant leg forward, non-weight-bearing foot rests on an elevated bench (~40–50 cm); front knee flexes to ~90° before returning to standing.	Dominant	1 × 6	40 s		
		3. Box Step-up	Participant steps onto an elevated box (~30–40 cm) leading with the non-dominant leg, driving through the heel to full hip/knee extension, then steps down under control.	Non-dominant	3 × 12	40 s		
		4. Single-leg Calf Raise	Standing on the dominant leg, participant performs full plantarflexion (heel raise) and controlled return to the starting position.	Dominant	1 × 12	40 s		
	— 5-min recovery period separating resistance and plyometric blocks —					—	5	
	Plyometric	1. Lunge Jump	Forward horizontal jump taking off and landing on the non-dominant leg; landing impact absorbed via controlled knee/hip flexion.	Non-dom Both limbs	3 × 12	60 s	12	Bodyweight resistance; maximal voluntary effort under coach supervision
		2. Single-leg Hop with Back-foot Raise	Standing on the dominant leg with the contralateral leg flexed behind the body; repeated vertical hops performed while maintaining single-leg support.	Dominant Both limbs	1 × 12	60 s		
		3. Single-leg Lateral Jump	Repeated side-to-side hops on the non-dominant leg over a marked lateral distance (~40–50 cm), emphasizing frontal-plane control and rapid ground contact.	Non-dominant Both limbs	3 × 12	60 s		
		4. Single-leg Continuous Hopping	Repeated forward hops on the dominant leg with minimal ground-contact time between hops (reactive/rebound-style hopping).	Dominant Both limbs	1 × 12	60 s		
CG	Regular handball training only (no additional unilateral resistance or plyometric training).							

EG = Experimental Group; CG = Control Group.

Note: The training protocol remained unchanged throughout the six-week intervention, with no planned progression in sets, repetitions, exercise selection, or external load, consistent with the original protocol described by Zhang et al. [22]. All exercises were performed using bodyweight resistance only (no external load), with participants instructed to perform each repetition with maximal voluntary effort under coach supervision. Perceived exertion was not formally quantified using a validated rating scale.

Table S2. Results of the Shapiro-Wilk and Levene Tests

Variables	Shapiro-Wilk Test (p value)				Levene's Test (p value)	
	Pre Test		Post Test			
	EG	CG	EG	CG	Pre Test	Post Test
Body Fat (%)	0,579	0,532	0,665	0,481	0,080	0,160
Body Fat (kg)	0,569	0,140	0,766	0,067	0,100	0,210
Body Muscle (%)	0,085	0,060	0,453	0,075	0,605	0,081
Body Muscle (kg)	0,525	0,384	0,571	0,232	0,394	0,379
Right Ext (Nm)	0,430	0,221	0,148	0,213	0,835	0,983
Right Flex (Nm)	0,447	0,800	0,183	0,550	0,272	0,639
Left Ext (Nm)	0,415	0,963	0,184	0,940	0,950	0,354
Left Flex (Nm)	0,393	0,129	0,872	0,055	0,053	0,411
20-m Sprint (s)	0,058	0,514	0,717	0,555	0,945	0,710
COD (s)	0,156	0,071	0,470	0,512	0,104	0,290
CMJ (cm)	0,737	0,067	0,507	0,147	0,075	0,081
SL – CMJ_R (cm)	0,581	0,066	0,226	0,253	0,352	0,869
SL – CMJ_L (cm)	0,576	0,283	0,052	0,617	0,703	0,350
Bilateral Q (%)	0,339	0,129	0,053	0,339	0,346	0,194
Bilateral H (%)	0,412	0,234	0,086	0,178	0,857	0,101
H/Q – Right (%)	0,259	0,130	0,105	0,372	0,437	0,608
H/Q – Left (%)	0,496	0,714	0,122	0,512	0,212	0,329
